# Automatic Analysis of Lateral Cephalograms Based on Multiresolution Decision Tree Regression Voting

**DOI:** 10.1155/2018/1797502

**Published:** 2018-11-19

**Authors:** Shumeng Wang, Huiqi Li, Jiazhi Li, Yanjun Zhang, Bingshuang Zou

**Affiliations:** ^1^School of Information and Electronics, Beijing Institute of Technology, Beijing, China; ^2^Department of Oral Health Sciences, Faculty of Dentistry, University of British Columbia, Vancouver, Canada; ^3^Viterbi School of Engineering, University of Southern California, Los Angeles, USA

## Abstract

Cephalometric analysis is a standard tool for assessment and prediction of craniofacial growth, orthodontic diagnosis, and oral-maxillofacial treatment planning. The aim of this study is to develop a fully automatic system of cephalometric analysis, including cephalometric landmark detection and cephalometric measurement in lateral cephalograms for malformation classification and assessment of dental growth and soft tissue profile. First, a novel method of multiscale decision tree regression voting using SIFT-based patch features is proposed for automatic landmark detection in lateral cephalometric radiographs. Then, some clinical measurements are calculated by using the detected landmark positions. Finally, two databases are tested in this study: one is the benchmark database of 300 lateral cephalograms from 2015 ISBI Challenge, and the other is our own database of 165 lateral cephalograms. Experimental results show that the performance of our proposed method is satisfactory for landmark detection and measurement analysis in lateral cephalograms.

## 1. Introduction

Cephalometric analysis is a scientific research approach for assessment and prediction of craniofacial growth, orthodontic diagnosis, and oral-maxillofacial treatment planning for patients with malocclusion in clinical practice [1]. We focus on 2D lateral cephalometric analysis, which is performed on cephalometric radiographs in lateral view. Cephalometric analysis has undergone three stages of development: manual stage, computer-aided stage, and computer-automated stage. Cephalometric analysis based on radiographs was introduced by Broadbent [2] and Hofrath [3] for the first time in 1931. In the first stage, it consists of five steps to obtain the cephalometric analysis: (a) placing a sheet of acetate over the cephalometric radiograph; (b) manual tracing of craniofacial anatomical structures; (c) manual marking of cephalometric landmarks; (d) measuring angular and linear parameters using the landmark locations; and (e) analysis/classification of craniomaxillofacial hard tissue and soft tissue [4]. This process is tedious, time consuming, and subjective. In the second stage, the first step in traditional cephalometric analysis has been skipped since the cephalometric radiograph is digitized. Furthermore, the next two steps can be operated by computer, and the measurement can be automatically calculated by software. However, this computer-aided analysis is still time consuming and the results are not reproducible due to large inter- and intravariability error in landmark annotation. In the third stage, the most crucial step, i.e., identifying landmarks, can be automatized by image processing algorithms [5]. The automatic analysis has high reliability and repeatability, and it can save a lot of time for the orthodontists. However, fully automatic cephalometric analysis is challenging due to overlaying structures and inhomogeneous intensity in cephalometric radiographs as well as anatomical differences among subjects.

Cephalometric landmarks include corners, line intersections, center points and other salient features of anatomical structures. They always have stable geometrical locations in anatomical structures in cephalograms. In this study, 45 landmarks are used in lateral cephalograms (refer to Table 1). The cephalometric landmarks play a major role in calculating the cephalometric planes, which are the lines between two landmarks in 2D cephalometric radiographs as shown in Table 2. Furthermore, different measurements are calculated using different cephalometric landmarks and planes, and they are used to analyze different anatomical structures [4]. Measurements can be angles or distance, which are used to analyze skeletal and dental anatomical structures, as well as soft tissue profile. To conduct a clinical diagnosis, many analytic approaches have been developed, including Downs analysis, Wylie analysis, Riedel analysis, Steiner analysis, Tweed analysis, Sassouni analysis, Bjork analysis, and so on [6].

Methods of automatic cephalometric landmark detection are mainly separated into three categories: (1) bottom-up methods; (2) deformation model-based methods; and (3) classifier/regressor-based methods. The first category is bottom-up methods, while the other two categories are learning-based methods.

For bottom-up methods, two techniques were usually employed, including edge detection and template matching. Edge-based methods were to extract the anatomical contours in cephalograms, and then the relative landmarks were identified on the contours using prior knowledge. Levy-Mandel et al. [7] first proposed the edge tracking method for identifying craniofacial landmarks. First, input images were smoothed by median filter and then edges were extracted by Mero-Vassy operator. Second, contours are obtained by the edge tracking technique based on the constraints of locations, endings, and breaking segments and edge linking conditions. Finally, the landmarks are detected on contours according to their definition in cephalometry. This method was only tested by two high quality cephalometric radiographs. Furthermore, 13 landmarks on noncontours were not detected. A two-stage landmark detection method was proposed by Grau et al. [8], which first extracted the major line features in high contrast by line-detection module, such as contours of jaws and nose, and then detected landmarks by pattern recognition based on mathematical morphology. Edge-based methods could only detect the landmarks on contours and were not robust to noise, low contrast, and occlusion. Template-matching-based methods were to find a most likely region with the least distance to the template of the specific landmark, and the center of the region was considered as the estimated position of the specific landmark. Therefore, not only the landmarks on contours, but also the landmarks on noncontours can be detected. Ashish et al. [9] proposed a template-matching method using a coarse-to-fine strategy for cephalometric landmark detection. Mondal et al. [10] proposed an improved method from Canny edge extraction to detect the craniofacial anatomical structures. Kaur and Singh [11] proposed an automatic cephalometric landmark detection using Zernike moments and template matching. Template matching based methods had difficulty in choosing the representative template and were not robust to anatomical variability in individual. All these methods strongly depended on the quality of images.

Subsequently, deformation model-based methods were proposed for these limitations by using shape constraints. Forsyth and Davis [12] reviewed and evaluated the approaches reported from 1986 to 1996 on automatic cephalometric analysis systems, and they highlighted a cephalometric analysis system presented by Davis and Taylor, which introduced an appearance model into landmark detection. The improved algorithms of active shape/appearance models were then used to refine cephalometric landmark detection combining with template matching, and higher accuracy was obtained [13–17]. Here, shape or appearance models were learned from training data to regularize the searching through all landmarks in testing data. However, it was difficult to initialize landmark positions for searching, because the initialization was always achieved by traditional methods.

Recently, significant progress has been made for automatic landmark detection in cephalograms by using supervised machine-learning approaches. These machine-learning approaches can be further separated into two classes: classification and regression models. A support vector machine (SVM) classifier was used to predict the locations of landmarks in cephalometric radiographs, while the projected principal-edge distribution was proposed to describe edges as the feature vector [18]. El-Feghi et al. [19] proposed a coarse-to-fine landmark detection algorithm, which first used fuzzy neural network to predict the locations of landmarks, and then refined the locations of landmarks by template matching. Leonardi et al. [20] employed the cellular neural networks approach for automatic cephalometric landmark detection on softcopy of direct digital cephalometric X-rays, which was tested by 41 cephalograms and detected 10 landmarks. Favaedi et al. [21] proposed a probability relaxation method based on shape features for cephalometric landmark detection. Farshbaf and Pouyan et al. [22] proposed a coarse-to-fine SVM classifier to predict the locations of landmarks in cephalograms, which used histograms of oriented gradients for coarse detection and histograms of gray profile for refinement. Classifier-based methods were successfully used to identify the landmark from the whole cephalograms, but positive and negative samples were difficult to be balanced in training data, and computational complexity is increased due to pixel-based searching.

Many algorithms have been reported for automatic cephalometric landmark detection, but results were difficult to compare due to the different databases and landmarks. The situation has been better since two challenges were held at 2014 and 2015 IEEE International Symposium on Biomedical Imaging (ISBI). During the challenges, regressor-based methods were first introduced to automatic landmark detection in lateral cephalograms. The challenges aimed at automatic cephalometric landmark detection and using landmark positions to measure the cephalometric linear and angular parameters for automatic assessment of anatomical abnormalities to assist clinical diagnosis. Benchmarks have been achieved by using random forest (RF) [23] regression voting based on shape model matching at the challenges. In particular, Lindner et al. [24, 25] presented the algorithm of random forest regression voting (RFRV) in the constrained local model (CLM) framework for automatic landmark detection, which obtained the mean error of 1.67 mm, and the successful detection rate of 73.68% in precision range of 2 mm. The algorithm of RFRV-CLM won the challenges, and the RF-based classification algorithm combined with Haar-like appearance features and game theory [26] was in rank 2. The comprehensive performance analysis among those algorithms for the challenges were reported in [27, 28]. Later, Vandaele et al. [29] proposed an ensemble tree-based method using multiresolution features in bioimages, and the method was tested by three databases including a cephalogram database of 2015 ISBI challenge. Now, public database and evaluation are available to improve the performance of automatic analysis system for cephalometric landmark detection and parameter measurement, and many research outcomes have been achieved. However, automatic cephalometric landmark detection and parameter measurement for clinical practice is still challenging.

**Figure 1:**
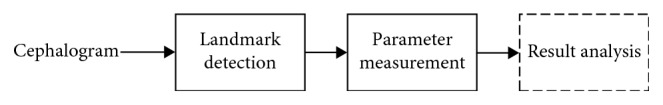
Block diagram of automatic cephalometric analysis system.

In recent days, efforts have been made to develop automatic cephalometric analysis systems for clinical usages. In this paper, we present a new automatic cephalometric analysis system, including landmark detection and parameter measurement in lateral cephalograms, and the block diagram of our system is shown in Figure 1. The core of this system is automatic landmark detection, which is realized by a new method based on multiresolution decision tree regression voting. Our main contributions can be concluded in four aspects: (1) we propose a new landmark detection framework of multiscale decision tree regression voting; (2) SIFT-based patch feature is first employed to extract the local feature of cephalometric landmarks, and the proposed approach is flexible when extending to detection of more landmarks because feature selection and shape constraints are not used; (3) two clinical databases are used to evaluate the extension of the proposed method. Experimental results show that our method can achieve robust detection when extending from 19 landmarks to 45 landmarks; (4) automatic measurement of clinical parameters is implemented in our system based on the detected landmarks, which can facilitate clinical diagnosis and research.

The rest of this paper is organized as follows.  Section 2 describes our proposed method of automatic landmark detection based on multiscale decision tree regression voting and parameter measurement. Experimental results for 2015 ISBI Challenge cephalometric benchmark database and our own database are presented in Section 3. The discussion of the proposed system is given in Section 4. Finally, we conclude this study with expectation of future work in Section 5.

## 2. Method

### 2.1. Landmark Detection

It is well known that cephalometric landmark detection is the most important step in cephalometric analysis. In this paper, we propose a new framework of automatic cephalometric landmark detection, which is based on multiresolution decision tree regression voting (MDTRV) using patch features.

#### 2.1.1. SIFT-Based Patch Feature Extraction

*(1). SIFT Feature*. Scale invariant feature transform (SIFT) is first proposed for key point detection by Lowe [30]. The basic idea of SIFT feature extraction algorithm consists of four steps: (1) local extrema point detection in scale-space by constructing the difference-of-Gaussian image pyramids; (2) accurate key point localization including location, scale, and orientation; (3) orientation assignment for invariance to image rotation; and (4) key point descriptor as the local image feature. The advantage of SIFT features to represent the key points is affine invariant and robust to illumination change for images. This method of feature extraction has been commonly used in the fields of image matching and registration.

*(2) SIFT Descriptor for Image Patch*. Key points with discretized descriptors can be used as visual words in the field of image retrieval. Histogram of visual words can then be used by a classifier to map images to abstract visual classes. Most successful image representations are based on affine invariant features derived from image patches. First, features are extracted on sampled patches of the image, either using a multiresolution grid in a randomized manner, or using interest point detectors. Each patch is then described using a feature vector, e.g., SIFT [30]. In this paper, we use the SIFT feature vectors [31] to represent image patches, which can be used by a regressor to map patches to the displacements from each landmark.

**Figure 2:**
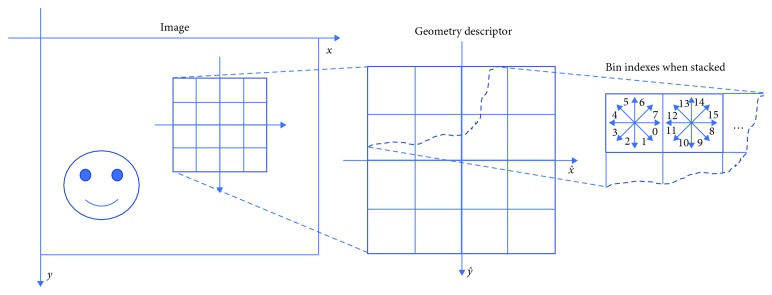
Diagram of SIFT feature descriptor of an image patch.

The diagram of SIFT feature descriptor of an image patch is illustrated in Figure 2. An image patch centered at location (*x*, *y*) will be described by a square window of length 2*W* + 1, where *W* is a parameter. For each square image patch *P* centered at position (*x*, *y*) of length 2*W* + 1, the gradient *P*_*x*_ and *P*_*y*_ of image patch *P* is computed by using finite differences. The gradient magnitude *g*_m_ is computed by

(1) $$\begin{array}{c} g_{\text{m}}=\sqrt{P_{x}^{2}+P_{y}^{2}}, \end{array}$$

and the gradient angle *g*_a_ (measured in radians, clockwise, starting from the *X* axis) is calculated by

(2) $$\begin{array}{c} g_{\text{a}}=\arctan\frac{P_{y}}{P_{x}}. \end{array}$$

**Figure 3:**
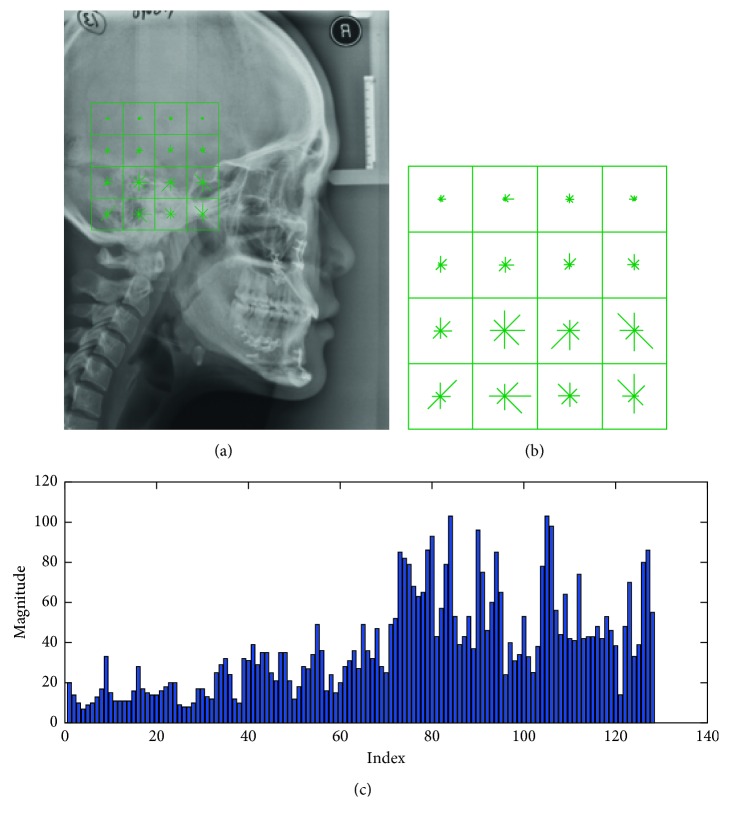
SIFT-based patch feature extraction. (a) Original image showing a patch. (b) Geometry descriptor. (c) The extracted feature vector.

Each square window is separated into 4 × 4 adjacent small windows, and the 8-bin histogram of gradients (direction angles started from 0 to (2π) is extracted in each small window. For each image patch, 4 × 4 histograms of gradients are concatenated to the resulting feature vector **f** (dimension = 128). Finally, the feature **f** of image patch *P* can be described as SIFT descriptor. The example of SIFT-based patch feature extraction in a cephalogram is shown in Figure 3.

#### 2.1.2. Decision Tree Regression

Decision tree is a classical and efficient statistical learning algorithm and has been widely used to solve classification and regression problems [32, 33]. Decision trees predict responses to data. To predict a response, query the new data by the decisions from the root node to a leaf node in the tree. The leaf node contains the response. Classification trees give responses that are nominal, while regression trees give numeric responses. In this paper, CART (classification and regression trees) [34], a binary tree, is used as the regressors *R*_*l*_ to learn the mapping relationship between the SIFT-based patch feature vectors *f* and the displacement vectors **d**(*dx*, *dy*) from the centers of the patches to the position of each landmark in the training images. The regressors *R*_*l*_ are then used to predict the displacements **d** using patch feature vectors of the test image. Finally, the predicted displacements are used to obtain the optimal location of each landmark via voting. One advantage of the regression approach is to avoid balancing positive and negative examples for the training of classifiers. Another advantage of using regression, rather than classification, is that good results can be obtained by evaluating the region of interest on randomly sampling pixels rather than at every pixel.

*(1) Training*. In training process, a regression tree *R*_*l*_ can be constructed for each landmark *l* via splitting. Here, optimization criterion and stopping rules are used to determine how a decision tree is to grow. The decision tree can be improved by pruning or selecting the appropriate parameters.

The optimization criterion is to choose a split to minimize the mean-squared error (MSE) of predictions compared to the ground truths in the training data. Splitting is the main process of creating decision trees. In general, four steps are performed to split node *t*. First, for each observation *f*_*j*_ (i.e., the extracted feature in the training data), the weighted MSE *ε*_*t*_ of the responses (displacements **d**) in node *t* is computed by using

(3) $$\begin{array}{c} \varepsilon_{t}=\underset{j\in T}{\sum }\frac{{\left(d_{j}-\bar{d_{t}}\right)}^{2}}{N},\;j=1,\ldots ,N, \end{array}$$

where *T* is the set of all observation indices in node *t* and *N* is the sample size. Second, the probability of an observation in node *t* is calculated by

(4) $$\begin{array}{c} P\left(T\right)=\underset{j\in T}{\sum }w_{j}, \end{array}$$

where *w*_*j*_ is the weight of observation *j*. In this paper, set *w*_*j*_ = 1/*N*. Third, all elements of the observation are sorted in ascending order. Every element is regraded as a splitting candidate. *T*_U_ is the unsplit set of all observation indices corresponding to missing values. Finally, the best way to split node *t* using *f*_*j*_ is determined by maximizing the reduction in MSE Δ*ε*_*t*_ among all splitting candidates. For all splitting candidates in the observation *f*_*j*_, the following steps are performed:

(1) Split the observations in node *t* into left and right child nodes (*t*_L_ and *t*_R_, respectively).
(2) Compute the reduction in MSE Δ*ε*_*t*_. For a particular splitting candidate, *t*_L_ and *t*_R_ represent the observation indices in the sets *T*_L_ and *T*_R_, respectively. If *f*_*j*_ does not contain any missing values, then the reduction in MSE Δ*ε*_*t*_ for the current splitting candidate is
  (5) $$\begin{array}{c} \Delta \varepsilon_{t}=P\left(T\right)\varepsilon_{t}-P\left(T_{\text{L}}\right)\varepsilon_{t_{\text{L}}}-P\left(T_{\text{R}}\right)\varepsilon_{t_{\text{R}}}. \end{array}$$

If *f*_*j*_ contains any missing values, then the reduction in MSE Δ*ε*_*t*_′ is
  (6) $$\begin{array}{c} \Delta \varepsilon_{t}^{\prime }=P\left(T-T_{\text{U}}\right)\varepsilon_{t}-P\left(T_{\text{L}}\right)\varepsilon_{t_{\text{L}}}-P\left(T_{\text{R}}\right)\varepsilon_{t_{\text{R}}}, \end{array}$$
  where *T* − *T*_U_ is the set of all observation indices in node *t* that are not missing.

(3) Choose the splitting candidate that yields the largest MSE reduction. In this way, the observations in node *t* are split at the candidate that maximize the MSE reduction.

To stop splitting nodes of the decision tree, two rules can be followed: (1) it is pure of the node that the MSE for the observed response in this node is less than the MSE for the observed response in the entire data multiplied by the tolerance on quadratic error per node and (2) the decision tree reaches to the setting values for depth of the regression decision tree, for example, the maximum number of splitting nodes *max_splits*.

The simplicity and performance of a decision tree should be considered to improve the performance of the decision tree at the same time. A deep tree usually achieves high accuracy on the training data. However, the tree is not to obtain high accuracy on a test data as well. It means that a deep tree tends to overfit, i.e., its test accuracy is much less than its training accuracy. On the contrary, a shallow tree does not achieve high training accuracy, but can be more robust, i.e., its training accuracy could be similar to that of a test data. Moreover, a shallow tree is easy to interpret and saves time for prediction. In addition, tree accuracy can be obtained by cross validation, when there are not enough data for training and testing.

There are two ways to improve the performance of decision trees by minimizing cross-validated loss. One is to select the optimal parameter value to control depth of decision trees. The other is postpruning after creating decision trees. In this paper, we use the parameter *max_splits* to control the depth of resulting decision trees. Setting a large value for *max_splits* lends to growing a deep tree, while setting a small value for *max_splits* yields a shallow tree with larger leaves. To select the appropriate value for *max_splits*, the following steps are performed: (1) set a spaced set of values from 1 to the total sample size for *max_splits* per tree; (2) create cross-validated regression trees for the data using the setting values for *max_splits* and calculate the cross-validated errors; and (3) the appropriate value of *max_splits* can be obtained by minimizing the cross-validated errors.

*(2) Prediction*. In prediction process, you can easily predict responses for new data after creating a regression tree *R*_*l*_. Suppose **f_new** is the new data (i.e., a feature vector extracted from a new patch *P_new* in the test cephalogram). According to the rules of the regression tree, the nodes select the specific attributes from the new observation **f_new** and reach the leaf step by step, which stores the mean displacement **d_new**. Here, we predict the displacement from the center of patch *P_new* to the landmark *l* using SIFT feature vector by the regressor *R*_*l*_.

#### 2.1.3. MDTRV Using SIFT-Based Patch Features

**Figure 4:**
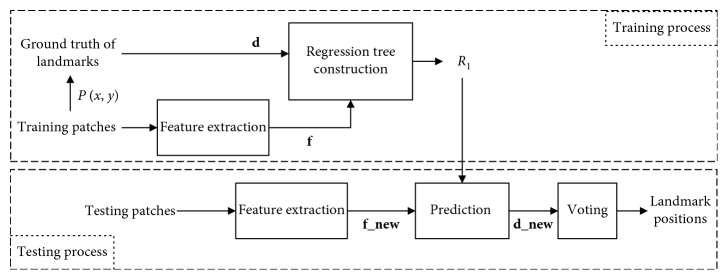
Algorithm of decision tree regression voting.

*(1) Decision Tree Regression Voting (DTRV)*. As illustrated in Figure 4, the algorithm of automatic cephalometric landmark detection using decision tree regression voting (DTRV) in single scale consists of training and testing processes as a supervised learning algorithm. The training process begins by feature extraction for patches sampled from the training images. Then, a regression tree is constructed for each landmark with inputting feature vectors and displacements (**f**, **d**). Here, **f** represents the observations and **d** represents the targets for this regression problem. The testing process begins by the same step of feature extraction. Then, the resulting **f_new** is used to predict the displacement **d_new** by regressor *R*_*l*_. In the end, the optimal landmark position is obtained via voting. The voting style includes single unit voting and single weighted voting. As mentioned in [35], these two styles perform equally well in application of voting optimal landmark positions for medical images. In this paper, we use the single unit voting.

*(2) MDTRV Using SIFT-Based Patch Features*. Finally, a new framework of MDTRV using SIFT-based patch features is proposed by using a simple and efficient strategy for accurate landmark detection in lateral cephalograms. There are four stages in the proposed algorithm of MDTRV, which are iteratively performed in the scales of 0.125, 0.25, 0.5, and 1. When the scale is 0.125, the training *K* patches with *K* displacements are randomly sampled in a whole cephalogram for a specific landmark *l*(*l*=1,…, *L*). The decision tree regressor *R*_*l*_^1^ is created by using these training samples. For prediction, SIFT features are extracted for *K*′ testing patches sampled in the whole cephalogram, and *K*′ displacements are predicted by regressor *R*_*l*_^1^ using extracted features. The optimal position of the specific landmark *l* is obtained via voting through all predicted displacements. When the scale is 0.25, 0.5, or 1, only the patch sampling rule is different from the procedure in scale of 0.125. That is, the training and testing patches are randomly sampled in the (2*S* + 1) × (2*S* + 1) neighborhood of true and initial landmark positions. The estimated landmark position is used as the initial landmark position in the next scale of testing process. The optimal position of the specific landmark *l* in scale of 1 is refined by using Hough forest [36], which is regarded as the final resulting location. This multiresolution coarse-to-fine strategy has greatly improved the accuracy of cephalometric landmark detection.

### 2.2. Parameter Measurement

Measurements are either angular or linear parameters calculated by using cephalometric landmarks and planes (refer to Tables 1 and 2). According to geometrical structure, measurements can be classified into five classes: the angle of three points, the angle of two planes, the distance between two points, the distance from a point to a plane, and the distance between two points projected to a plane. All measurements can be calculated automatically in our system as described in the following.

#### 2.2.1. The Angle of Three Points

Assume point B is the vertex among three points A, B, and C, then the angle of these three points is calculated by

(7) $$\begin{array}{c} ∠ABC=\arccos\;\;\left(\frac{a^{2}+c^{2}-b^{2}}{2ac}\right)\times \left(\frac{180}{\pi }\right), \end{array}$$

and

(8) $$\begin{array}{c} \left\{\begin{array}{c} a=\left\|A-B\right\|,\\ b=\left\|A-C\right\|,\\ c=\left\|B-C\right\|, \end{array}\right. \end{array}$$

where $\left\|A-B\right\|=\sqrt{{\left(x_{A}-x_{B}\right)}^{2}+{\left(y_{A}-y_{B}\right)}^{2}}$ represents the Euclidean distance of *A* and *B*.

#### 2.2.2. The Angle between Two Planes

As the cephalometric radiographs are 2D images in this study, the planes are projected as straight lines. Thus, the planes are determined by two points. The angle between two planes $\bar{AB}$ and $\bar{CD}$ is calculated by

(9) $$\begin{array}{c} \left\{\begin{array}{c} ∠\bar{AB}=\arctan\;\;\left(\frac{y_{A}-y_{B}}{x_{A}-x_{B}}\right)\times \left(\frac{180}{\pi }\right),\\ ∠\bar{CD}=\arctan\;\;\left(\frac{y_{C}-y_{D}}{x_{C}-x_{D}}\right)\times \left(\frac{180}{\pi }\right), \end{array}\right. \end{array}$$

(10) $$\begin{array}{c} ∠\bar{ABCD}=\left|∠\bar{AB}-∠\bar{CD}\right|. \end{array}$$

#### 2.2.3. The Distance between Two Points

The distance between two points is calculated using the following equation:

(11) $$\begin{array}{c} \left|AB\right|=\left\|A-B\right\|\times \text{ratio,} \end{array}$$

ratio is a scale indicator that can be calculated by the calibrated gauge in the lateral cephalograms and its unit is mm/pixel.

#### 2.2.4. The Distance from a Point to a Plane in the Horizontal Direction

The plane $\bar{AB}$ is defined as

(12) $$\begin{array}{c} y+k_{1}x+k_{0}=0. \end{array}$$

Thus, the distance of point C to the plane $\bar{AB}$ is calculated by

(13) $$\begin{array}{c} \left|C\cdot \bar{AB}\right|=\left|\frac{y_{C}+k_{1}x_{C}+k_{0}}{\sqrt{1+k_{1}^{2}}}\right|\times \text{ratio}. \end{array}$$

#### 2.2.5. The Distance between Two Points Projected to a Plane

**Figure 5:**
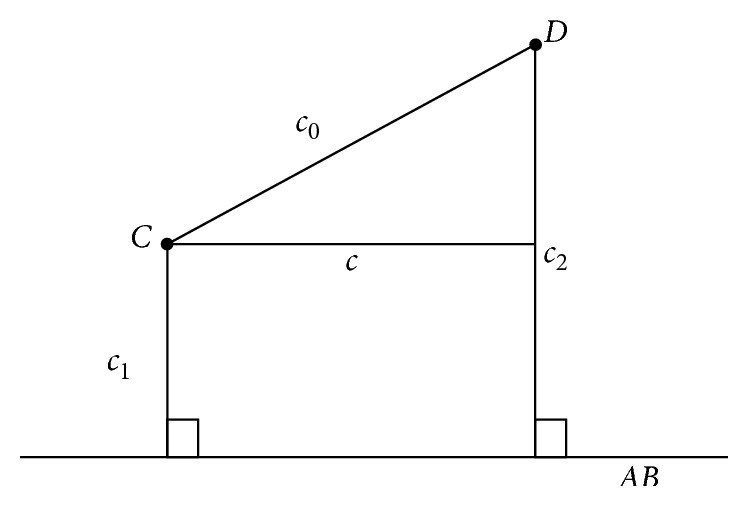
The distance calculation between two points projected to a plane.

The calculation of the distance between two points projected to a plane is illustrated in Figure 5. In order to calculate the distance between two points projected to a plane, first we use Equations (4)–(12) to determine the plane $\bar{AB}$. Then, we calculate the distance from two points *C* and *D* to the plane $\bar{AB}$ as *c*_1_ and *c*_2_ by Equations (4)–(13). Third, the distance *c*_0_ between two points C and D is calculated by Equations (4)–(11). Finally, the distance $\left|C\cdot \bar{AB}\cdot D\right|$ between two points *C* and *D* projected to the plane $\bar{AB}$ is represented as *c* and is calculated by

(14) $$\begin{array}{c} c=\sqrt{c_{0}^{2}-{\left(c_{1}-c_{2}\right)}^{2}}\times \text{ratio}. \end{array}$$

## 3. Experimental Evaluation

### 3.1. Data Description

#### 3.1.1. Database of 2015 ISBI Challenge

**Figure 6:**
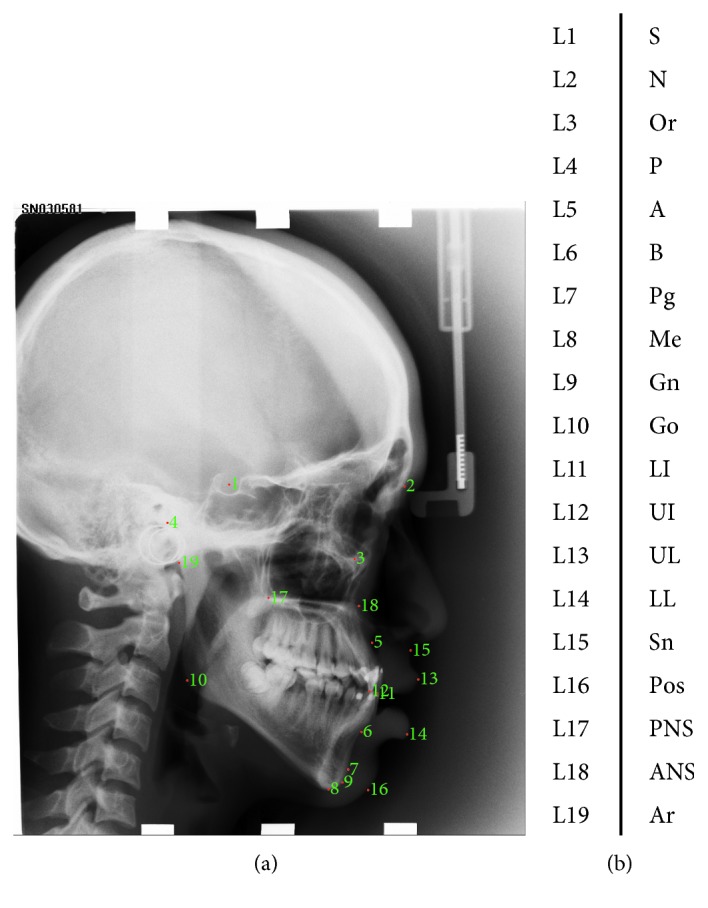
Cephalogram annotation example showing the 19 landmarks in database1. (a) Cephalogram annotation example. (b) Description of 19 landmarks.

The benchmark database (database1) included 300 cephalometric radiographs (150 for TrainingData, 150 for Test1Data), which is described in Wang et al. [28]. All of the cephalograms were collected from 300 patients aged from 6 to 60 years old. The image resolution was 1935 × 2400 pixels. For evaluation, 19 landmarks were manually annotated by two experienced doctors in each cephalogram (see illustrations in Figure 6); the ground truth was the average of the annotations by both doctors, and eight clinical measurements were used for classification of anatomical types.

#### 3.1.2. Database of Peking University School and Hospital of Stomatology

**Figure 7:**
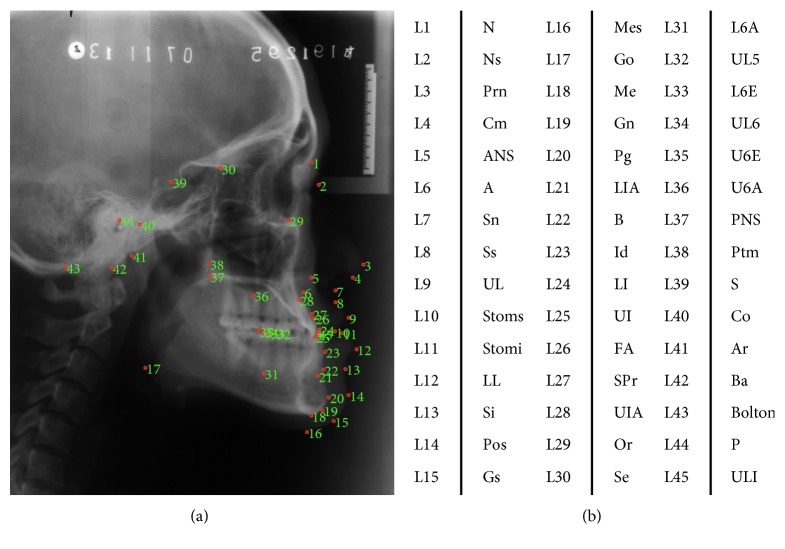
Cephalogram annotation example showing landmarks in database2. (a) Cephalogram annotation example. (b) The description of 45 cephalometric landmarks.

The database2 included 165 cephalometric radiographs as illustrated in Table 3 (the IRB approval number is PKUSSIRB-201415063). There were 55 cephalograms collected from 55 Chinese adult subjects (29 females, 26 males) who were diagnosed as skeletal class I (0 < ANB < 4) with minor dental crowding and a harmonious. The other 110 cephalograms were collected from the 55 skeletal class III patients (ANB < 0) (32 females, 23 males), who underwent combined surgical orthodontic treatment at the Peking University School and Hospital of Stomatology from 2010 to 2013. The image resolutions were different in the range from 758 × 925 to 2690 × 3630 pixels. For evaluation, 45 landmarks were manually annotated by two experienced orthodontists in each cephalogram as shown in Figure 7; the ground truth was the average of annotations by both doctors, and 27 clinical measurements were used for future cephalometric analysis.

#### 3.1.3. Experimental Settings

In the initial scale of landmark detection, for training, *K* = 50; for prediction, *K*′ = 400. In the other scales, the additional parameters *W* and *S* are set to 48 and 40, separately. Because the image resolutions are different, preprocessing is required to rescale all the images to the fixed width of 1960 pixels in database2. For database2, we test our algorithm by using 5-fold cross validation.

### 3.2. Landmark Detection

#### 3.2.1. Evaluation Criterion

The first evaluation criterion is the mean radial error with the associated standard deviation. The radial error *E*, i.e., the distance between the predicted position and the true position of each landmark, is defined as

(15) $$\begin{array}{c} E_{i}=\left\|A_{i}-B_{i}\right\|\times \text{ratio},\;a_{i}\in A,b_{i}\in B,1\leq i\leq M, \end{array}$$

where *A*, *B* represent the estimated and true positions of each landmark for all cephalograms in the dataset; *A*_*i*_, *B*_*i*_ are the corresponding positions in set *A* and *B*, respectively; and *M* is the total number of cephalograms in the dataset. The mean radial error (MRE) and the associated standard deviation (SD) for each landmark are defined as

(16) $$\begin{array}{c} \text{MRE}=\text{mean}\left(E\right)=\frac{\sum_{i=1}^{M}E_{i}}{M},\\ \text{SD}=\sqrt{\frac{\sum_{i=1}^{M}{\left(E_{i}-\text{MRE}\right)}^{2}}{M-1}.} \end{array}$$

The second evaluation criterion is the success detection rate with respect to the 2.5 mm, 3 mm, 5 mm, and 10 mm precision ranges. If *E*_*i*_ is less than a precision range, the detection of the landmark is considered as a successful detection in the precision range; otherwise, it is considered as a failed detection. The success detection rate (SDR) *p*_*z*_ with precision less than *z* is defined as

(17) $$\begin{array}{c} p_{z}=\frac{\left\{E_{i}<z\right\}}{M}\times 100\%,\;1\leq i\leq M, \end{array}$$

where *z* denotes four precision ranges used in the evaluation, including 2.0 mm, 2.5 mm, 3 mm, and 4 mm.

#### 3.2.2. Experimental Results

*(1) Results of database1*. Experimental results of cephalometric landmark detection using database1 is shown in Table 4. The MREs of landmarks L10, L4, L19, L5, and L16 are more than 2 mm. The other 14 landmarks are all within the MRE of 2 mm, in which 3 landmarks are within the MRE of 1 mm. The average MRE and SD of 19 landmarks are 1.69 mm and 1.43 mm, respectively. It shows that the detection of cephalometric landmarks is accurate by our proposed method. The SDRs are 73.37%, 79.65%, 84.46%, and 90.67% within the precision ranges of 2.0, 2.5, 3.0, and 4.0 mm, respectively. In 2 mm precision range, the SDR is more than 90% for four landmarks (L9, L12, L13, L14); the SDR is between 80% and 90% for six landmarks (L1, L7, L8, L11, L15, L17); the SDR is between 70% and 80% for two landmarks (L6 and L18); the SDRs for the other seven landmarks are less than 70%, where the SDR of landmark L10 is the lowest. It can be seen from Table 4 that the landmark L10 is the most difficult to detect accurately.

Comparison of our method with three state-of-the-art methods using Test1data is shown in Table 5. The difference between MRE of our proposed method and RFRV-CLM in [24] is less than 1 pixel, which means that their performance is comparable within the resolution of image.

Furthermore, we conduct the comparison with the top two methods in 2015 ISBI Challenge of cephalometric landmark detection in terms of MRE, SD, and SDR as illustrated in Table 6. Our method achieves the SDR of 73.37% within the precision range of 2.0 mm, which is similar to the SDR of 73.68% of the best method. The MRE of our proposed method is 1.69 mm, which is comparable to that of method of RFRV-CLM, but the SD of our method is less than that of method of RFRV-CLM. The results show that our method is accurate for landmark detection in lateral cephalograms and is more robust.

**Figure 8:**
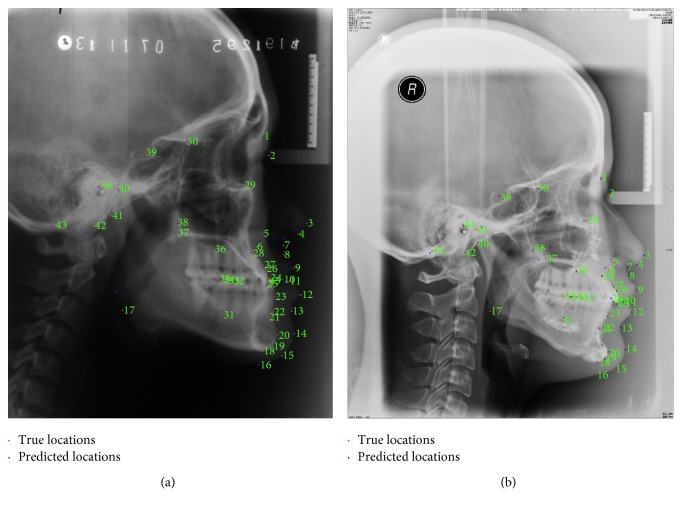
Examples of automatic cephalometric landmark detection. (a) Example of detection in Class I. (b) Example of detection in Class III.

*(2) Results of database2*. Two examples of landmark detection in database2 are shown in Figure 8. It can be observed from the figure that the predicted locations of 45 landmarks in cephalograms are near to the ground truth locations, which shows the success of our proposed algorithm for landmark detection.

For the quantitative assessment of the proposed algorithm, the statistical result is shown in Table 7. The MRE and SD of the proposed method to detect 45 landmarks are 1.71 mm and 1.39 mm, respectively. The average SDRs of 45 landmarks within the precision range 2.0 mm, 2.5 mm, 3 mm, and 4 mm are 72.08%, 80.63%, 86.46%, and 93.07%, respectively. The experimental results in database2 show comparable performance to the results of database1. It indicates that the proposed method can be successfully applied to the detection of more clinical landmarks.

### 3.3. Measurement Analysis

#### 3.3.1. Evaluation Criterion

For the classification of anatomical types, eight cephalometric measurements were usually used. The description of these eight measurements and the methods for classification are explained in Tables 8 and 9, respectively.

One evaluation criterion for measurement analysis is the success classification rate (SCR) for these 8 popular methods of analysis, which is calculated using confusion matrix. In the confusion matrix, each column represents the instances of an estimated type, while each row represents the instances of the ground truth type. The SCR is defined as the averaged diagonal of the confusion matrix.

For evaluation, the performance of the proposed system for more measurement analysis in database2, another criterion, the mean absolute error (MAE), is calculated by

(18) $$\begin{array}{c} \text{MAE}=\frac{\sum_{i=1}^{M}\left|{\hat{V}}_{i}-V_{i}\right|}{M}, \end{array}$$

where ${\hat{V}}_{i}$ is the value of angular or linear measurement estimated by our system and *V*_*i*_ is the ground truth measurement obtained using human annotated landmarks.

#### 3.3.2. Experimental Results

*(1) Results of database1*. Using the detected 19 landmarks, 8 cephalometric measurements are calculated for classification of anatomical types in database1. The comparison of our method to the best two methods is given in Table 10, where we have achieved the best classification results for two measurements of APDI and FHI. The average SCR obtained by our method is 75.03%, which is much better than the method in [26] and is comparable to the method of RFRV-CLM [24].

*(2) Results of database2*. According to the detected 45 landmarks, 27 measurements are automatically calculated by our system using database2, including 17 angular and 10 linear measurements, which are illustrated in Table 11.

The MAE and MAE^*∗*^ of 27 measurements are illustrated in Table 12. Here, the MAE represents the performance of measurement analysis of our automatic system. The MAE^*∗*^ represents interobserver variability calculated between the ground truth values obtained by the two experts. For angular measurements, the difference between MAE and MAE^*∗*^ is within 0.5° for 9/17 measurements; the difference between MAE and MAE^*∗*^ is within 1° for 12/17 measurements, and the difference between MAE and MAE^*∗*^ is within 2° for 16/17 measurements. In particular, the MAE of *Z* Angle is less than MAE^*∗*^. The other one measurement has the difference of 2.24°. For linear measurements, the difference between MAE and MAE^*∗*^ is within 0.5 mm for 9/10 measurements. The other one measurement has the difference of 1.16 mm. The results show that our automatic system is efficient and accurate for measurement analysis.

## 4. Discussion

The interobserver variability of human annotation is analyzed.  Table 13 shows that the MRE and SD between annotations by two orthodontists are 1.38 mm and 1.55 mm for database1, respectively [27]; for database2, the MRE and SD between annotations of two orthodontists are 1.26 mm and 1.27 mm, respectively. Experimental results show that the proposed algorithm of automatic cephalometric landmark detection can achieve the MRE of less than 2 mm and the SD almost equal to that of manual marking. Therefore, the performance of automatic landmark detection by the proposed algorithm is comparable to manual marking in term of the interobserver variability between two clinical experts. Furthermore, the detected landmarks are used to calculate the angular and linear measurements in lateral cephalograms. The satisfactory results of measurement analysis are presented in experiments based on the accurate landmark detection. It shows that the proposed algorithm has the potential to be applied in clinical practice of cephalometric analysis for orthodontic diagnosis and treatment planning.

The detection accuracy of some landmarks is lower than the average value, and there are mainly three main reasons: (i) some landmarks are located at the overlaying anatomical structures, such as landmarks Go, UL5, L6E, UL6, and U6E; (ii) some landmarks have large variability of manual marking due to large anatomical variability among subjects especially in abnormality, such as landmarks A, ANS, Pos, Ar, Ba, and Bolton; and (iii) structural information is not obvious due to little intensity variability in the neighborhood of some landmarks in images, such as landmarks P, Co, L6A, and U6A.

The proposed system follows clinical cephalometric analysis procedure, and the accuracy of the system can be evaluated by the manual marking accuracy. There are several limitations in this study. On one hand, except for the algorithm, the data effects to the performance of the system include three aspects: (i) the quality of the training data; (ii) the size of the training dataset; and (iii) the shape and appearance variation exhibited in the training data. On the other hand, performance of the system depends on consistency between the training data and the testing data, similarly as any supervised learning-based methods.

## 5. Conclusion

In conclusion, we design a new framework of landmark detection in lateral cephalograms with low-to-high resolutions. In each image resolution, decision tree regression voting is employed in landmark detection. The proposed algorithm takes full advantage of image information in different resolutions. In lower resolution, the primary local structure information rather than local detail information can be extracted to predict the positions of anatomical structure involving the specific landmarks. In higher resolution, the local structure information involves more detail information, and it is useful for prediction positions of landmarks in the neighborhood. As demonstrated in experimental results, the proposed algorithm has achieved good performance. Compared with state-of-the-art methods using the benchmark database, our algorithm has obtained the comparable accuracy of landmark detection in terms of MRE, SD, and SDR. Tested by our own clinical database, our algorithm has also obtained average 72% successful detection rate within precision range of 2.0 mm. In particular, 45 landmarks have been detected in our database, which is over two times of the number of landmarks in the benchmark database. Therefore, the extensibility of the proposed algorithm is confirmed using this clinical dataset. In addition, automatic measurement of clinical parameters has also achieved satisfactory results. In the future, we will put more efforts to improve the performance of automatic analysis in lateral cephalograms so that the automatic system can be utilized in clinical practice to obtain objective measurement. More research will be conducted to reduce the computational complexity of the algorithm as well.

## Figures and Tables

**Table 1:** Description of cephalometric landmarks.

Symbol	Description
N	Nasion
Ns	Soft tissue nasion
Prn	Pronasale
Cm	Columella
ANS	Anterior nasal spine
A	Subspinale
Sn	Subnasale
Ss/A′	Upper pit of lips
UL	Upper lip
Stoms	Stomion superius
Stomi	Stomion inferius
LL	Lower lip
Si/B′	Lower pit of lips
Pos	Soft tissue pogonion
Gs	Soft tissue gnathion
Mes	Soft tissue menton
Go	Gonion
Me	Menton
Gn	Gnathion
Pg	Pogonion
LIA	Mandibular joint midpoint
B	Supramental
Id	Infradentale
LI	Lower incisal incision
UI	Upper incisal incision
FA	Maxillary incisor's facial axis
SPr	Superior prosthion
UIA	Root point of incisor
Or	Orbitale
Se	Internode of sphenoid wing with anterior cranial fossa
L6A	Root apex of mandibular first molar
UL5	Midpoint of tip of the second molar
L6E	Buccal apex of mandibular first molar
UL6	Midpoint of tip of first molar
U6E	Buccal apex of maxillary first molar
U6A	Root apex of maxillary first molar
PNS	Posterior nasal spine
Ptm	Pterygomaxillary fissure
S	Sella
Co	Condylion
Ar	Articulare
Ba	Basion
Bolton	Concave point of posterior incision of occipital condyle
P	Porion
ULI	Midpoint of lower incisor and upper incisor

**Table 2:** Description of cephalometric planes.

Name	Involving landmarks	Description in database1	Description in database2
*Reference planes*			
SN. Anterior cranial base plane	S-N	$\bar{\text{L}1\text{L}2}$	$\bar{\text{L}39\text{L}1}$
FH. Frankfort horizontal plane	P-O	$\bar{\text{L}4\text{L}3}$	$\bar{\text{L}44\text{L}29}$
Bolton plane	Bolton-N	—	$\bar{\text{L}43\text{L}1}$
*Measurement planes*			
Palatal plane	ANS-PNS	$\bar{\text{L}18\text{L}17}$	$\bar{\text{L}5\text{L}37}$
Cranial base plane	Ba-N	—	$\bar{\text{L}42\text{L}1}$
MP. Mandibular plane	Go-Me	$\bar{\text{L}10\text{L}8}$	$\bar{\text{L}17\text{L}18}$
RP. Ramal plane	Ar-Go	$\bar{\text{L}19\text{L}10}$	$\bar{\text{L}41\text{L}17}$
NP. Facial plane	N-Pg	$\bar{\text{L}2\text{L}7}$	$\bar{\text{L}1\text{L}20}$
NA plane	N-A	$\bar{\text{L}2\text{L}5}$	$\bar{\text{L}1\text{L}6}$
AB. Subspinale to infradentale plane	A-B	$\bar{\text{L}5\text{L}6}$	$\bar{\text{L}6\text{L}22}$
AP plane	A-Pg	$\bar{\text{L}5\text{L}7}$	$\bar{\text{L}6\text{L}20}$
*Soft tissue measurement planes*			
Facial plane of soft tissue	Ns-Pos	—	$\bar{\text{L}2\text{L}14}$
Ricketts esthetic plane	Prn-Pos	—	$\bar{\text{L}3\text{L}14}$
H plane	UL-Pos	$\bar{\text{L}13\text{L}16}$	$\bar{\text{L}9\text{L}14}$

**Table 3:** The description of database2.

		Female	Male
Class I		29	26
Class III	Pretreatment	32	23
	Posttreatment	32	23
Total number of cephalograms		165	
Total number of patients		110	

**Table 4:** The experimental results of 19 landmark detection in Test1Data.

Landmark	MRE (mm)	SD (mm)	SDR (%)			
			2.0 mm	2.5 mm	3.0 mm	4.0 mm
L1	1.31	1.26	86.67%	92.67%	94.67%	97.33%
L2	1.92	2.05	68.00%	72.00%	79.33%	88.67%
L3	1.81	1.74	66.67%	81.33%	88.00%	95.33%
L4	3.11	2.59	50.00%	54.00%	59.33%	67.33%
L5	2.24	1.56	56.00%	66.00%	75.33%	86.67%
L6	1.59	1.39	72.00%	78.67%	84.00%	94.00%
L7	1.23	0.87	80.67%	91.33%	96.67%	99.33%
L8	1.08	0.88	88.67%	95.33%	96.67%	98.67%
L9	0.87	0.75	91.33%	93.33%	98.00%	99.33%
L10	3.98	2.33	23.33%	29.33%	38.00%	53.33%
L11	0.97	0.89	87.33%	92.00%	96.00%	98.67%
L12	0.90	1.70	94.67%	94.67%	96.00%	96.67%
L13	1.02	0.71	92.00%	95.33%	98.00%	100.00%
L14	0.89	0.61	93.33%	98.67%	100.00%	100.00%
L15	1.18	1.16	85.33%	92.00%	93.33%	98.00%
L16	2.14	1.48	50.00%	60.67%	72.67%	90.67%
L17	1.16	0.85	86.00%	92.67%	94.67%	98.67%
L18	1.77	1.51	72.00%	79.33%	86.00%	92.67%
L19	2.97	2.77	50.00%	54.00%	58.00%	67.33%
Average	1.69	1.43	73.37%	79.65%	84.46%	90.67%

**Table 5:** Comparison of our method with three methods in term of MRE using Test1Dtata.

Method	[24]	[26]	[29]	Ours
MRE (pixels)	16.74	18.46	17.79	16.92

**Table 6:** Comparison of our method to two methods in terms of MRE, SD, and SDR using Test1Dtata.

Method	MRE (mm)	SD (mm)	SDR (%)			
			2.0 mm	2.5 mm	3.0 mm	4.0 mm
[24]	1.67	1.65	73.68%	80.21%	85.19%	91.47%
[26]	1.84	1.76	71.72%	77.40%	81.93%	88.04%
Ours	1.69	1.43	73.37%	79.65%	84.46%	90.67%

**Table 7:** The statistical results of automatic cephalometric landmark detection in database2.

No. of 5-fold	MRE (mm)	SD (mm)	SDR (%)			
			2.0 mm	2.5 mm	3.0 mm	4.0 mm
1	1.72	1.35	72.49%	81.32%	87.04%	93.37%
2	1.72	1.38	71.38%	79.94%	85.63%	92.73%
3	1.66	1.32	74.31%	82.06%	87.44%	93.37%
4	1.77	1.36	69.67%	78.96%	85.32%	92.93%
5	1.68	1.54	72.53%	80.88%	86.87%	92.96%
Average	1.71	1.39	72.08%	80.63%	86.46%	93.07%

**Table 8:** Description of cephalometric measurements in database1 [28].

No.	Measurement	Description in mathematics	Description in words
1	ANB	∠L5L2L6	The angle between the landmark 5, 2, and 6
2	SNB	∠L1L2L6	The angle between the landmark 1, 2, and 6
3	SNA	∠L1L2L5	The angle between the landmark 1, 2, and 5
4	ODI	$∠\bar{\text{L}5\text{L}6\text{L}8\text{L}10}+∠\bar{\text{L}17\text{L}18\text{L}4\text{L}3}$	The arithmetic sum of the angle between AB plane ($\bar{\text{L}5\text{L}6}$) to mandibular plane ($\bar{\text{L}8\text{L}10}$) and the angle of palatal plane ($\bar{\text{L}17\text{L}18}$) to FH plane ($\bar{\text{L}4\text{L}3}$)
5	APDI	$∠\bar{\text{L}3\text{L}4\text{L}2\text{L}7}+∠\bar{\text{L}2\text{L}7\text{L}5\text{L}6}+∠\bar{\text{L}3\text{L}4\text{L}17\text{L}18}$	The arithmetic sum of the angle between FH plane ($\bar{\text{L}3\text{L}4}$) to facial plane ($\bar{\text{L}2\text{L}7}$), the angle of facial plane ($\bar{\text{L}2\text{L}7}$) to AB plane ($\bar{\text{L}5\text{L}6}$), and the angle of FH plane ($\bar{\text{L}3\text{L}4}$) to palatal plane ($\bar{\text{L}17\text{L}18}$)
6	FHI	$\left|\bar{\text{L}1\text{L}10}\right|/\left|\bar{\text{L}2\text{L}8}\right|$	The ratio of posterior face height (PFH, the distance from L1 to L10) to anterior face height (AFH, the distance from L2 to L8)
7	FHA	$∠\bar{\text{L}1\text{L}2\text{L}10\text{L}9}$	The angle between SN plane ($\bar{\text{L}1\text{L}2}$) to mandibular plane ($\bar{\text{L}10\text{L}9}$)
8	MW	$\left\{\begin{array}{cc} \left|\bar{\text{L}12\text{L}11}\right|, & x\left(\text{L}12\right)<x\left(L11\right),\\ -\left|\bar{\text{L}12\text{L}11}\right|, & \text{otherwise}. \end{array}\right.$	When the *x* ordinate of L12 is less than L11's, the MW is $\left|\bar{\text{L}12\text{L}11}\right|$; otherwise, MW is $-\left|\bar{\text{L}12\text{L}11}\right|$.

**Table 9:** Eight standard cephalometric measurement methods for classification of anatomical types [28].

No.	Measurement	Type 1	Type 2	Type 3	Type 4
1	ANB	3.2°∼5.7° class I (normal)	>5.7° class II	<3.2° class III	—
2	SNB	74.6°∼78.7° normal mandible	<74.6° retrognathic mandible	>78.7° prognathic mandible	—
3	SNA	79.4°∼83.2° normal maxilla	>83.2° prognathic maxilla	<79.4° retrognathic maxilla	—
4	ODI	Normal: 74.5° ± 6.07°	>80.5° deep bite tendency	<68.4° open bite tendency	—
5	APDI	Normal: 81.4° ± 3.8°	<77.6° class II tendency	>85.2° class III tendency	—
6	FHI	Normal: 0.65∼0.75	>0.75 short face tendency	<0.65 long face tendency	—
7	FHA	Normal: 26.8°∼31.4°	>31.4° mandible high angle tendency	<26.8° mandible lower angle tendency	—
8	MW	Normal: 2 mm∼4.5 mm	MW = 0 mm edge to edge	MW <0 mm anterior cross bite	MW >4.5 mm large over jet

**Table 10:** Comparison of our method to two methods in term of SCR using Test1Data.

Method	The success classification rates, SCR (%)								
	ANB	SNB	SNA	ODI	APDI	FHI	FHA	MW	Average
[24]	64.99%	84.52%	68.45%	84.64%	82.14%	67.92%	75.54%	82.19%	76.30%
[26]	59.42%	71.09%	59.00%	78.04%	80.16%	58.97%	77.03%	83.94%	70.96%
Ours	**58.61%**	**78.85%**	**59.86%**	**76.59%**	**83.49%**	**82.44%**	**77.18%**	**83.20%**	**75.03%**

**Table 11:** Description of cephalometric measurements in database2.

Name	Description in mathematics	Description in words
*The angles of three points (unit: °)*		
SNA	∠L39L1L6	The angle of landmarks L39, L1, L6
SNB	∠L39L1L22	The angle of landmarks L39, L1, L22
ANB	∠L6L1L22	The angle of landmarks L6, L1, L22
SNPg	∠L39L20L1	The angle of landmarks L39, L20, L1
NAPg	∠L1L6L20	The angle of landmarks L1, L6, L20
NSGn	∠L1L39L19	The angle of landmarks L1, L39, L19
Ns-Prn-Pos	∠L2L3L14	The angle of landmarks L2, L3, L14
Cm-Sn-UL	∠L4L7L9	The angle of landmarks L4, L7, L9
LL-B′-Pos	∠L12L13L14	The angle of landmarks L12, L13, L14
Angle of the jaw	∠L41L17L18	The angle of landmarks L41, L17, L18
Angle of convexity	∠L2L7L14	The angle of landmarks L2, L7, L14
*The angles between two planes (unit: °)*		
FH-SN	$∠\bar{\text{L}44\text{L}29\text{L}39\text{L}1}$	The angle between FH plane ($\bar{\text{L}44\text{L}29}$) and SN plane ($\bar{\text{L}39\text{L}1}$)
UI-SN	$∠\bar{\text{L}28\text{L}25\text{L}39\text{L}1}$	The angle between upper incisor ($\bar{\text{L}28\text{L}25}$) and SN plane ($\bar{\text{L}39\text{L}1}$)
LI-FH	$∠\bar{\text{L}24\text{L}21\text{L}44\text{L}29}$	The angle between lower incisor axis ($\bar{\text{L}24\text{L}21}$) and FH plane ($\bar{\text{L}44\text{L}29}$)
UI-LI	$∠\bar{\text{L}28\text{L}25\text{L}24\text{L}21}$	The angle between upper and lower incisors ($\bar{\text{L}28\text{L}25}$, $\bar{\text{L}24\text{L}21}$)
H angle	$∠\bar{\text{L}9\text{L}14\text{L}8\text{L}14}$	The angle between H plane ($\bar{\text{L}9\text{L}14}$)and NB plane ($\bar{\text{L}8\text{L}14}$)
Z angle	$∠\bar{\text{L}44\text{L}29\text{L}9\text{L}14}$	The rear lower angle between FH plane ($\bar{\text{L}44\text{L}29}$) and H plane ($\bar{\text{L}9\text{L}14}$)
*The distances between two points (unit: mm)*		
N-Me	$\left|\bar{\text{L}1\text{L}18}\right|$	The forward height, the distance between landmarks L1, L18
N-ANS	$\left|\bar{\text{L}1\text{L}5}\right|$	The up-forward height, the distance between landmarks L1, L5
ANS-Me	$\left|\bar{\text{L}5\text{L}18}\right|$	The down-forward height, the distance between landmarks L5, L18
Stoms-UI	$\left|\bar{\text{L}10\text{L}25}\right|$	The vertical distance between landmarks L10, L25
*The distances from the point to the plane in the horizontal direction (unit: mm)*		
UI-AP	$\left|\text{L}25\bar{\text{L}6\text{L}20}\right|$	The distance between landmark L25 to AP plane ($\bar{\text{L}6\text{L}20}$)
LI-AP	$\left|\text{L}24\bar{\text{L}6\text{L}20}\right|$	The distance from landmark L24 to AP plane ($\bar{\text{L}6\text{L}20}$)
UL-EP	$\left|\text{L}9\bar{\text{L}3\text{L}14}\right|$	The distance from landmark L9 to EP plane ($\bar{\text{L}3\text{L}14}$)
LL-EP	$\left|\text{L}12\bar{\text{L}13\text{L}14}\right|$	The distance from landmark L12 to EP plane ($\bar{\text{L}3\text{L}14}$)
Max.E	$\left|\text{L}6\bar{\text{L}5\text{L}37}\right|$	Maxillary length, the distance from landmark L6 to palatal plane ($\bar{\text{L}5\text{L}37}$)
*The distances between two points projected to a plane (unit: mm)*		
Wits	$\left|\text{L}6\bar{\text{L}32\text{L}34}\text{L}22\right|$	The distance between the two landmarks L6 and L22 projected to functional jaw plane ($\bar{\text{L}32\text{L}34}$)

**Table 12:** Result of our method for 27 measurements in terms of MAE and MAE^*∗*^ using database2.

Name	MAE	MAE^*∗*^	MAE-MAE^*∗*^
*The angles of three points (unit: °)*			
SNA	1.95	1.70	0.24
SNB	1.57	1.17	0.39
ANB	1.29	1.08	0.21
SNPg	1.59	1.20	0.39
NAPg	2.83	2.44	0.39
NSGn	1.42	0.96	0.47
Ns-Prn-Pos	1.68	1.10	0.58
Cm-Sn-UL	5.52	3.80	1.72
LL-B′-Pos	3.95	3.44	0.51
Angle of the jaw	3.20	1.66	1.54
Angle of convexity	2.67	1.86	0.81
*The angles between two planes (unit: °)*			
FH-SN	2.00	1.96	0.04
UI-SN	4.71	2.81	1.90
LI-FH	3.34	2.27	1.07
UI-LI	6.90	4.66	2.24
H angle	0.94	0.80	0.14
Z angle	1.69	1.86	-0.17
*The distances between two points (unit: mm)*			
N-Me	1.37	1.10	0.27
N-ANS	1.57	1.34	0.24
ANS-Me	1.06	0.96	0.10
Stoms-UI	0.75	0.42	0.33
*The distances from the point to the plane in the horizontal direction (unit: mm)*			
UI-AP	0.96	0.71	0.25
LI-AP	0.96	0.76	0.20
UL-EP	0.50	0.36	0.14
LL-EP	0.45	0.39	0.05
Max.E	2.06	1.79	0.27
*The distances between two points projected to a plane (unit: mm)*			
Wits	4.70	3.53	1.16

MAE^*∗*^: interobserver variability.

**Table 13:** The interobserver error of manual marking between doctor1 and doctor2.

	Interobserver variability	
	MRE (mm)	SD (mm)
database1	1.38	1.55
database2	1.26	1.27

## Data Availability

The database1 of 2015 ISBI Challenge is available at http://www.ntust.edu.tw/∼cweiwang/ISBI2015/challenge1/index.html. The database2 of Peking University School and Hospital of Stomatology cannot be made public available due to privacy concerns for the patients.
